# The safety and effectiveness of transforaminal lumbar interbody fusion (TLIF) surgery for the treatment of lumbar disc herniation

**DOI:** 10.3389/fsurg.2025.1598531

**Published:** 2025-05-13

**Authors:** Wei Wang, Zhao Guo, Lixin Yang, Jianning Liu, Zhiyong Li, Jiaqi Li, Hong Zhao, Tao Sun

**Affiliations:** ^1^ Department of Orthopedics, Hebei Medical University Third Hospital, Shijiazhuang, Hebei, China; ^2^ Department of Orthopedics, Affiliated Hospital of Hebei University, Baoding, Hebei, China; ^3^ Department of Eye Trauma, Hebei Medical University Third Hospital, Shijiazhuang, Hebei, China

**Keywords:** transforaminal lumbar interbody fusion, lumbar disc herniation, postoperative complications, symptoms recurrence, risk factors

## Abstract

**Background:**

The purpose of this study is to discuss the safety and effectiveness of transforaminal lumbar interbody fusion (TLIF) for the treatment of lumbar disc herniation.

**Methods:**

From August 2018 to December 2021, patients with lumbar disc herniation who received TLIF treatment were included in this study. Clinical data collected during both the preoperative period and the 2-year postoperative follow-up were analyzed. The correlations between preoperative clinical indicators and postoperative functional outcomes were modeled using both univariate regression and multivariable-adjusted analyses.

**Result:**

The study population comprised 547 consecutive cases (male: 261, 47.7%; female: 286, 52.3%). Stratified outcome analysis showed 458 patients (83.7%) attained optimal surgical recovery without detectable morbidity, contrasted with 89 cases (16.3%) manifesting postoperative complications. The univariate analysis of postoperative complications found that the recurrence of symptoms was related to body mass index (BMI), preoperative pain time, High-level segment, intraoperative bleeding volume and postoperative visual analog scale (VAS)-back. Postoperative hematoma was related to hypertension and wound drainage. Poor wound healing was related to BMI and Wound drainage volume. However, this study failed to find the related factors of wound infection. After binary logistic analysis of the above single factors, we found that BMI and preoperative pain time were independent risk factors for symptom recurrence, and BMI were independent risk factors for Poor wound healing.

**Conclusion:**

Transforaminal lumbar interbody fusion surgery can safely and effectively treat lumbar disc herniation.

## Introduction

Lumbar disc herniation, as a common spinal surgical disease, is particularly common in the elderly population, with its main clinical manifestations being lower back pain and sciatica (1, 2). Although most cases respond well to conservative treatment, there are still a few patients who require surgical decompression, and recent studies have shown that surgical treatment yields significant benefits in both the short and long term (3, 4). In the treatment of unilateral intervertebral disc herniation, microsurgery has become a common choice due to its ability to significantly reduce damage (5, 6). However, it is worth noting that minimally invasive surgical methods such as microscopy or endoscopy are not suitable for all cases. Specifically, studies have shown that minimally invasive techniques may not achieve satisfactory treatment outcomes in Carragee II and IV hernias, as these types of hernias are more prone to recurrent disc herniation and segmental instability (7, 8). In clinical practice, decompression surgery methods such as lumbar discectomy and vertebral fusion have been proven to effectively improve symptoms of lower back pain and sciatica (9, 10). For patients suffering from unilateral disc herniation, transforaminal lumbar interbody fusion (TLIF), a classic lumbar surgical technique, has proven to be highly effective in alleviating lower back pain and sciatica, and most patients can achieve significant therapeutic effects (11). However, patients undergoing lumbar disc surgery may encounter a range of complications, such as symptom recurrence, postoperative hematoma, wound infection, and delayed wound healing. How to avoid these complications is currently a major issue that needs to be addressed after lumbar disc surgery. In light of these findings, this study aims to evaluate the safety and effectiveness of transforaminal lumbar interbody fusion surgery for the treatment of lumbar disc herniation.

## Materials and methods

### Patients

A retrospective analysis was conducted on 547 patients who received TLIF treatment from August 2018 to December 2021. Obtaining informed consent from each patient, this study was approved by the Ethics Review Committee of our hospital and obtained a unique study registration identification number (the research registration number is 20241111).

**Figure 1:**
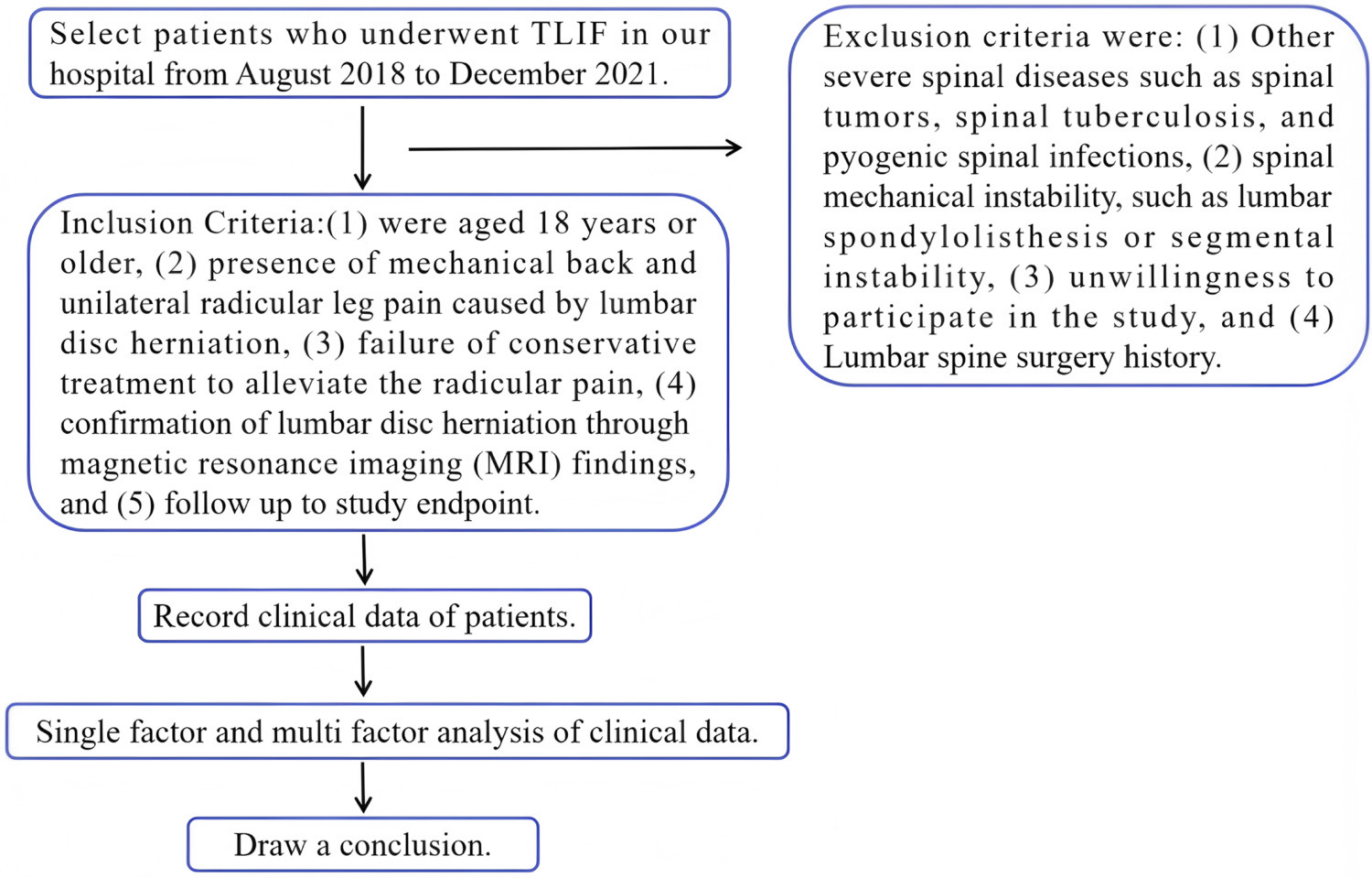
The research process of this study.

The inclusion criteria consisted of patients who: (1) were aged 18 years or older, (2) presence of mechanical back and unilateral radicular leg pain caused by lumbar disc herniation, (3) failure of conservative treatment to alleviate the radicular pain, (4) confirmation of lumbar disc herniation through magnetic resonance imaging (MRI) findings, and (5) follow up to study endpoint. Exclusion criteria were: (1) Other severe spinal diseases such as spinal tumors, spinal tuberculosis, and pyogenic spinal infections, (2) spinal mechanical instability, such as lumbar spondylolisthesis or segmental instability, (3) unwillingness to participate in the study, and (4) Lumbar spine surgery history. Figure 1 shows the research process of this study.

### Surgical technique

After general anesthesia, the patient is placed in a prone position with the abdomen suspended to reduce intra-abdominal pressure, thereby reducing bleeding from the venous plexus in the spinal canal during decompression operations. A midline incision on the lower back should be made, cutting through the skin and subcutaneous tissue. Then, the paraspinal muscles should be carefully removed from under the periosteum to reveal the vertebral plate and facet joints. Pedicle screws were placed, and then the lower articular process of one side of the upper vertebral body and the upper articular process of the lower vertebral body was bitten off by the vertebral plate clamp. When removing the ligamentum flavum, the dural sac is protected by the nerve peeler to prevent tearing the dura mater. The deep ligamentum flavum was removed and the lateral recess was decompressed. Then the hypertrophic ligamentum flavum and residual joint capsule were removed from the intervertebral foramen and lateral recess.

Expose the intervertebral disc from the intervertebral foramen area. Use a sharp knife to cut through the fibrous ring on the surface of the intervertebral disc. Use nucleus pulposus forceps to remove degenerated nucleus pulposus tissue from the intervertebral disc. Scrape off the upper and lower cartilage endplates, and gradually increase the height of the trial model to select the appropriate size of cage (Cage contacts the upper and lower vertebral plates). Wash the intervertebral space with sterile physiological saline, implant autologous bone, allogeneic bone fragments, and fusion cage and ensure the presence of bone tissue around the fusion cage. Place the appropriate length of the bowl rod and bending, and place the screw tail cap. Cover the surface of the dural sac with gelatin sponge. Place drainage tube and suture the layers.

### Clinical indexes

This study is to comprehensively collect and analyze clinical indicator data of cases, and track and record their follow-up results for up to 2 years after surgery. These include age, gender, osteoporosis, body mass index (BMI), painful limbs, hypertension, diabetes, occupation, lesion segment (multi segment, high-level segment), preoperative pain time, operation time, intraoperative blood loss, postoperative wound drainage, wound infection, deep vein thrombosis (DVT), imaging outcomes were assessed via lumbar x-rays and computed tomography (CT) at 3 months, 1 year, and 2 years postoperatively to evaluate fusion status (solid fusion vs. non-union) and cage position (subsidence defined as ≥2 mm vertical displacement or ≥10° angular deviation from the initial placement), preoperative visual analog scale (VAS)-back, postoperative VAS-back preoperative and postoperative oswestry disability index (ODI) scores and postoperative complications including symptoms recurrence, postoperative hematoma, wound infection and poor wound healing complications.

Osteoporosis was diagnosed with the aid of x-ray or computed tomography (CT) findings due to the infrequency of bone density testing in patients lumbar disc herniation (12, 13). In continuous variables, except for age and BMI, all other variables are dichotomized at the median as their high and low values. Obesity was defined as having a BMI of no less than 28. Advanced age is defined as the patient's age not less than 60 years old. Patients were divided into mental workers and manual workers according to their occupation. The patients who undertook a small amount of manual labor and worked mainly indoor were regarded as mental workers. They can also be called non-manual workers. The patients who undertook a large amount of manual labor and worked mainly outdoor were regarded as manual workers. Lesion segment was classified as “multi segment” if the lumbar disc herniation involved two or more adjacent spinal segments (e.g., L3–4 and L4–5), and “high-level segment” was defined as herniation occurring at the L1–2, L2–3 or L3–4 level, distinguishing it from the more common lower segments (L4–5 or L5-S1). DVT records did not include cases of lower extremity intermuscular thrombosis because this type of thrombosis was classified as a peripheral variant of DVT and essentially limited to the venous plexus of soleus and gastrocnemius. Studies have shown that lower extremity venous intermuscular thrombosis has almost no impact on patients (14). Symptom recurrence is manifested as the initial improvement of lower back pain and neuralgia caused by lumbar disc herniation after surgical treatment, followed by the recurrence of lower back pain and lower limb neuralgia associated with lumbar vertebrae (whether due to adjacent segment degeneration or contralateral foraminal stenosis) at 6 months postoperatively (15, 16). According to Centers for Disease Control and Prevention definition (17), Wound infection, including superficial wound infection, was defined as infection involving only the skin or subcutaneous tissue occurring within 30 days postoperatively, while deep wound infection was defined as infection occurring within a year post-surgery, confirmed to be operation-related, and involving deep soft tissues. In this research, no deep wound infection was found, so the wound infection refers to superficial wound infection. According to the process of wound healing (18), poor wound healing was diagnosed based on surgical wound rupture, scar hyperplasia, sinus formation, skin or flap necrosis and surgical wound exudation but there was no bacterial growth after 3 days of cultivation. Postoperative hematoma, also known as epidural hematoma, is the accumulation of blood in the spinal canal after surgery that compresses the cauda equina, nerve roots, or spinal cord that can result in devastating neurologic consequences and it was diagnosed by MRI and ultrasound.

### Perioperative management

Preoperatively, a comprehensive evaluation of the patient's cardiopulmonary function, coagulation mechanism, and nutritional status should be conducted. Additionally, for patients without contraindications, intramuscular injection of thrombin may be administered to help prevent intraoperative bleeding. Secondly, preoperative guidance is given to patients to perform axial turning to adapt to postoperative position limitations. At the same time, psychological counseling is used to alleviate patients' anxiety and enhance their confidence in treatment. During the operation, general anesthesia was chosen while monitoring the patient's vital signs. After surgery, the patient was given nebulization to assist in sputum discharge. Vital signs were continuously monitored within 24 h after surgery, and lung function exercise was initiated. Low molecular weight heparin was used 24 h after surgery to prevent lower limb thrombosis, and the patient was guided to turn over axially while keeping the wound dressing clean and dry.

### Postoperative rehabilitation exercise

Rehabilitation training is mainly aimed at training the core muscles of the lumbar spine. Specifically, within one day after surgery, actively perform ankle pump on the bed and passively perform straight leg lift test. From 1 to 7 days after surgery, actively engage in straight leg raise exercises. Wear an abdominal brace to provide support, and gradually begin to get out of bed and walk slowly, progressively increasing the walking distance over time. One week after surgery, gradually initiate the following exercises: five-point support, three-point support, plank support, and prone knee and hip flexion, among others. After discharge, the patient continued to complete the aforementioned exercises under the supervision of their family members until 3 months post-surgery and attended follow-up examinations as scheduled.

### Follow up and end point

Follow up of patients after discharge is necessary. Generally, patients are scheduled for regular follow-up examinations at one month, three months, six months, one year and two years after surgery. However, it is important to note if patients experience sudden situations such as significant back pain and lower limb neuralgia, they can come for diagnosis at any time. This study would have two endpoints. One is that during a 2-year period, the patient experienced severe back pain and lower limb neuralgia again and after being diagnosed with symptom recurrence through MRI examination, the time and VAS-back were recorded. The other is 2 years after surgery, at this point, all patients except those who have already completed the study would be evaluated.

### Statistics

To conduct a comparative analysis on patients' dissatisfaction as the dependent variable, we utilized a chi-square test for univariate analysis. Preoperative and postoperative ODI scores and VAS scores using t-test. All continuous variables are presented as mean ± standard deviation (SD). Variables that yielded a *P* value of less than 0.05 in the univariate analysis were included as input in the multivariate logistic regression model. For each variable, we computed the odds ratio (OR) with its 95% confidence interval (CI). In the chi-square test and multivariate logistic regression model, except for age and BMI, all the other continuous variables were dichotomized at the median. *P* value less than 0.05 was considered significant. All statistical analyses were done using SPSS software version 27.0 (SPSS, Inc., Chicago, IL, USA).

## Result

### General characteristics

A total of 547 patients who met the inclusion criteria from August 2018 to December 2021 were included in this study. Among them, there were 261 male patients (47.7%) and 286 female patients (52.3%). The average age and BMI were 55.2 years old and 26.89 kg/m², respectively. Among all patients, 51.7% (283/547) suffer from left lower limb pain and 48.3% (264/547) suffer from right lower limb pain. The average preoperative pain time is 5.7 months. Please refer to Table 1 for detailed basic information of patients.

**Table 1:** Basic information of 547 patients.

Parameters	Patients (*N*/%)
Gender	
Male	261 (47.7%)
Female	286 (52.3%)
Age (Mean ± SD)	55.1 ± 12.8
BMI (Mean ± SD)	26.89 ± 4.06
Occupation	
Mental workers	204 (37.3%)
Manual workers	343 (62.7%)
Hypertension	
Yes	150 (27.4%)
No	397 (72.6%)
Diabetes	
Yes	60 (11.0%)
No	487 (89.0%)
Osteoporosis	
Yes	60 (11.0%)
No	487 (89.0%)
Painful limbs	
Left	283 (51.7%)
Right	264 (48.3%)
Preoperative pain time in month (Mean ± SD)	5.7 ± 3.5
Preoperative VAS-back (Mean ± SD)	5.8 ± 1.1
Surgical segment	
Multi segment	47 (8.6)
High-level segment	21 (3.8)
Surgical time in minutes (Mean ± SD)	140.0 ± 31.1
Intraoperative bleeding volume (Mean ± SD)	588.6 ± 306.9
Wound drainage volume (Mean ± SD)	352.2 ± 188.8
Postoperative VAS-back (Mean ± SD)	1.6 ± 1.6
Postoperative thrombosis	
Yes	31 (5.7%)
No	516 (94.3%)

### Changes in ODI and VAS

We compared the preoperative and postoperative ODI scores and VAS scores of the collected data. The results revealed that both ODI and VAS scores demonstrated significant improvement after surgery compared to preoperative levels. This indicates that TLIF can effectively enhance patients' quality of life. The specific data is shown in Table 2.

**Table 2:** Comparison of VAS and ODI scores between preoperative and postoperative patients.

Time	VAS (Mean ± SD)	ODI (Mean ± SD)
Preoperative	5.8 ± 1.1	58.7 ± 6.3
Postoperative	1.6 ± 1.6	17.9 ± 11.0
*t*	65.996	76.326
*P*	<0.01	<0.01

### Survival analysis of symptom recurrence

**Figure 2:**
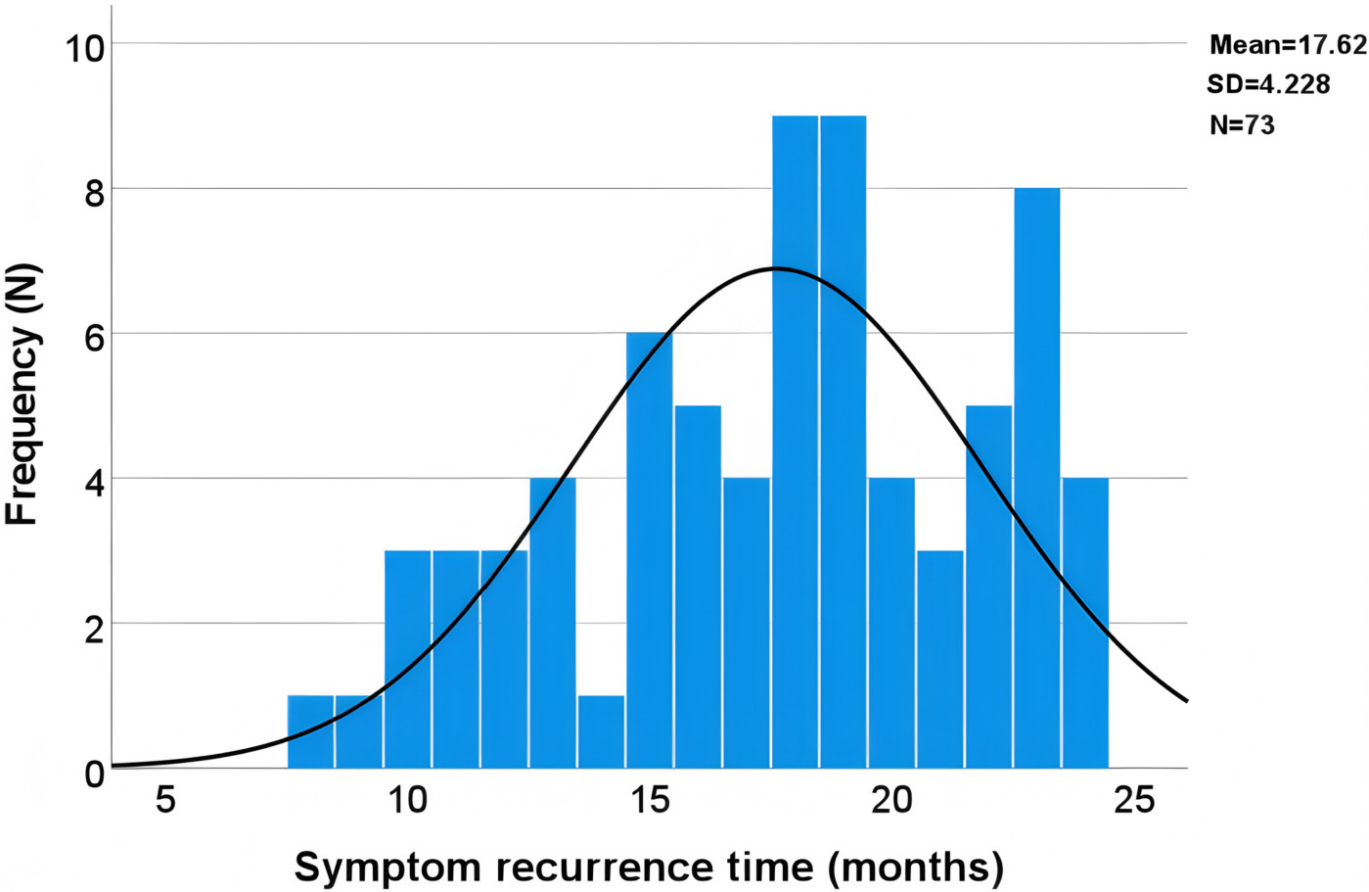
Time series distribution of symptom recurrence.

**Figure 3:**
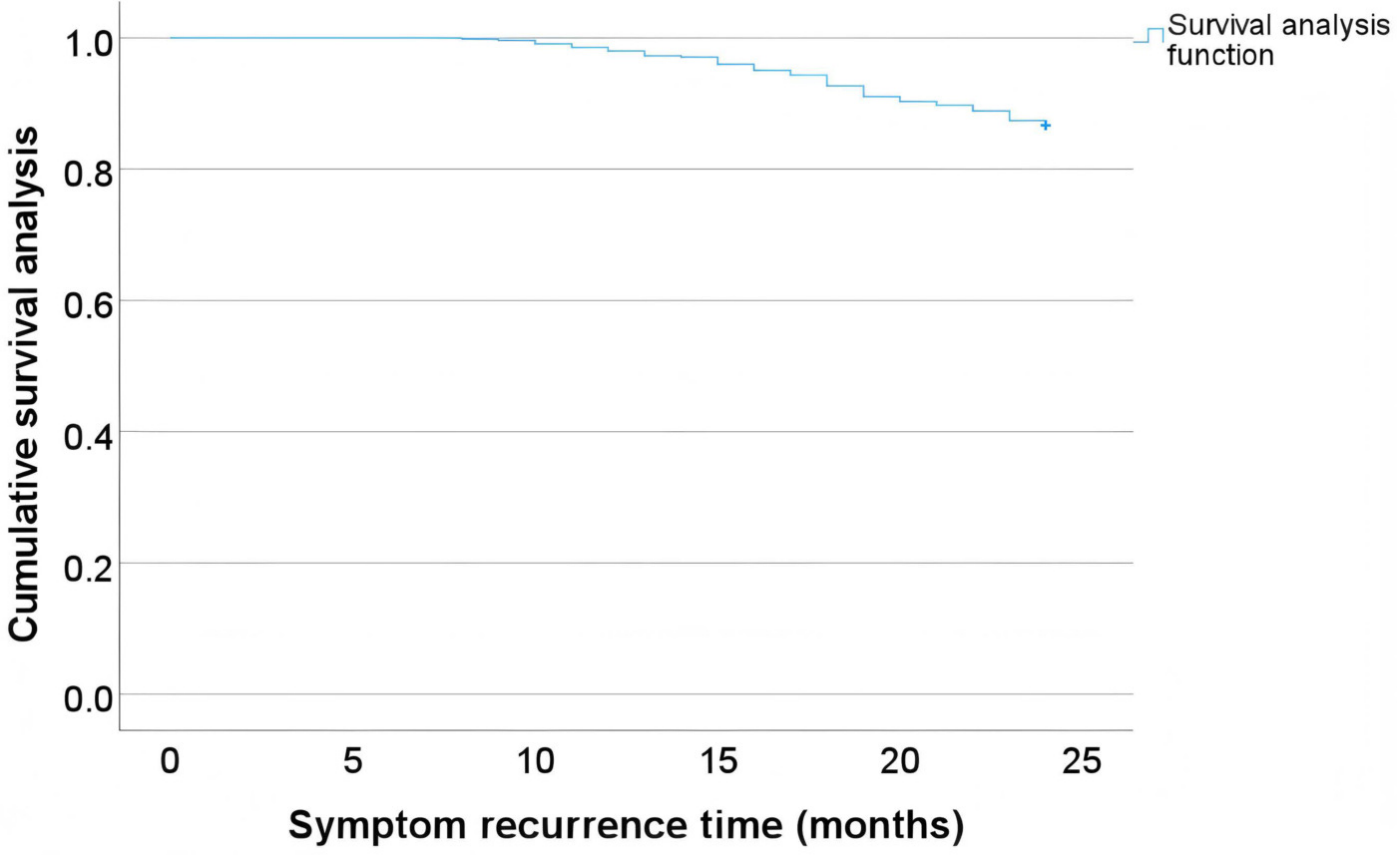
Survival analysis curve of symptom recurrence.

The most common complication, symptom recurrence, was analyzed using time series distribution plots to identify temporal patterns of postoperative symptom recurrence. Additionally, Kaplan-Meier survival analysis was employed to further assess the recurrence rates over time. By examining Figures 2,3 together, it is evident that the time of symptom recurrence in patients follows a normal distribution, with the peak incidence occurring at approximately 17.6 months post-surgery.

### Complication risk

Among all 547 patients, a total of 89 (16.3%) experienced complications and the incidence of postoperative complications in patients is shown in Table 3. In this study, the most common complication was symptom recurrence, which was seen in 13.3% (73/547) of patients. There were 4 patients who developed postoperative hematomas, all of which occurred within one week after surgery. After the occurrence of postoperative hematoma, patients underwent a second surgical debridement, and all recovered well. Four patients developed wound infections, and all four patients had their waist wounds contaminated with urine. Although dressing was changed promptly upon detection, infection ultimately occurred, and Escherichia coli was cultured in all wounds after secretion culture. The infected wound did not improve after dressing change, and after undergoing debridement surgery again, the wound ultimately achieved Grade A healing. This study reported a total of 8 patients who experienced poor wound healing. All 8 patients underwent regular dressing changes, wound disinfection, and enhanced suturing to achieve final healing. The wound ultimately achieved Grade A healing after suture removal. The results of the imaging evaluation showed that intervertebral fusion had reached a solid state in all cases, and there was displacement or angulation of the fusion cages over time.

**Table 3:** The incidence of postoperative complications in patients.

Postoperative complications	% (*N* = 547)
Total	16.3% (89/547)
Symptoms recurrence	13.3% (73/547)
Postoperative hematoma	0.7% (4/547)
Wound infection	0.7% (4/547)
Poor wound healing	1.5% (8/547)

The univariate analysis of postoperative complications found that the recurrence of symptoms was related to BMI, preoperative pain time, High-level segment, intraoperative bleeding volume and postoperative VAS-back. Postoperative hematoma was related to hypertension and wound drainage volume. Poor wound healing was related to BMI and Wound drainage volume. However, this study failed to find the related factors of wound infection. Table 4 shows the detailed relations between the adjusted univariate analysis parameters and complications.

**Table 4:** The relationship between single factor analysis parameters and complications.

Parameters	Postoperative complications (*X*^2^; *P*)			
	Symptoms recurrence	Postoperative hematoma	Wound infection	Poor wound healing
Gender	0.930; 0.335	0.008; 0.927	0.008; 0.927	0.017; 0.896
Age	1.650; 0.199	0.131; 0.718	0.131; 0.718	1.531; 0.216
BMI	23.829; **<0.001**	0.278; 0.598	0.260; 0.610	8.751; **0.003**
Occupation	0.840; 0.360	0.000; 0.995	0.000; 0.995	0.000; 0.993
Hypertension	0.077; 0.782	10.665; **0.001**	1.552; 0.217	2.079; 0.149
Diabetes	0.643; 0.423	0.496; 0.481	0.496; 0.481	1.637; 0.201
Osteoporosis	0.164; 0.685	0.496; 0.481	0.812; 0.367	1,637; 0.201
Painful limbs	3.311; 0.069	0.005; 0.944	0.873; 0.350	1.760; 0.185
Preoperative pain time in month	19.671; **<0.001**	0.914; 0.339	1.106; 0.293	1.842; 0.175
Preoperative VAS-back	1.484; 0.223	0.142; 0.706	0.415; 0.520	0.836; 0.361
Multi segment	3.710; 0.054	0.014; 0.907	1.536; 0.215	2.037; 0.153
High-level segment	4.309; **0.038**	0.721; 0.396	0.721; 0.396	0.609; 0.435
Surgical time in minutes	2.040; 0.153	0.073; 0.787	0.551; 0.458	0.112; 0.738
Intraoperative bleeding volume	6.065; **0.014**	0.337; 0.561	0.337; 0.561	1.863; 0.172
Wound drainage volume	0.608; 0.436	4.164; **0.041**	0.942; 0.332	4.771; **0.029**
Postoperative VAS-back	170.103; **<0.001**	0.193; 0.660	0.193; 0.660	1.170; 0.279
Postoperative thrombosis	0.437; 0.508	0.469; 0.494	0.469; 0.494	0.944; 0.331

Note: The bold data represents data with statistical significance in univariate analysis.

Perform multicollinearity tests on significant variables in univariate analysis using tolerance (TOL) and variance inflation factor (VIF). TOL > 0.10 or VIF < 10.0 indicates that there is no significant multicollinearity between variables. The results show that the TOL values range from 0.944–0.994 and the VIF values range from 1.006–1.060 among the variables. The variables can be included as predictive factors in the logistic regression model, and the specific results are shown in Table 5.

**Table 5:** Collinearity analysis of predictive variables for risk.

Factors	TOL	VIF
BMI	0.972	1.029
Preoperative pain time	0.967	1.034
High-level segment	0.994	1.006
Intraoperative bleeding volume	0.988	1.012
Postoperative VAS-back	0.944	1.060

After binary logistic analysis of the above single factors, we found that BMI and preoperative pain time were independent risk factors for symptom recurrence, and BMI were independent risk factors for poor wound healing. Seen Table 6 for details.

**Table 6:** Influencing factors of postoperative complications identified by binary logistic regression analysis.

Relevant factor	*B* value	Se value	Wald value	*P* value	*OR* value	95% *CI*
Symptoms recurrence						
BMI	0.977	0.313	9.754	0.002	2.655	1.439–4.901
Preoperative pain time in month	0.796	0.331	5.802	0.016	2.218	1.160–4.240
High-level segment	0.604	0.370	2.665	0.103	1.830	0.886–3.780
Intraoperative bleeding volume	0.362	0.332	1.190	0.275	1.437	0.794–2.756
Postoperative VAS-back	23.053	7,848.937	0.000	0.998	-	-
Postoperative hematoma						
Hypertension	−17.313	1,839.460	0.000	0.992	0.000	-
Wound drainage volume	−16.549	2,071.600	0.000	0.994	0.000	-
Poor wound healing						
BMI	−2.436	1.075	5.136	0.023	0.088	0.011–0.720
Wound drainage volume	−1.920	1.076	3.180	0.075	0.147	0.018–1.209

## Discussion

In this study, a total of 547 patients with lumbar disc herniation received TLIF treatment. Most patients did not experience any postoperative complications, only 89 patients (16.3%) experiencing complications including symptom recurrence, postoperative hematoma, wound infection, and poor wound healing, this is similar to other research findings, with a complication rate of 16.6% (19). Existing research indicates that although minimally invasive TLIF has the advantages of less intraoperative bleeding and shorter hospital stay. No significant differences in operative time and postoperative complications between the two surgeries were observed (20). There is sufficient evidence to suggest that we should identify preoperative and postoperative factors that affect postoperative complications in patients, in order to maximize treatment effectiveness. This study showed that BMI and preoperative pain time are independent risk factors for symptom recurrence, BMI is an independent risk factor for poor wound healing, and no related risk factor has been found for postoperative hematoma and wound infection. It is important to closely monitor these factors to improve the recovery effect in TLIF treatment of lumbar disc herniation.

This study found that obesity is a negative factor for patients undergoing TLIF surgery, whether it is related to poor postoperative wound healing or symptom recurrence. Li et al. found that 8.47% of all people had poor wound healing in their study of the effect of postoperative hypoalbuminemia and supplement of human serum albumin on the development of poor wound healing following lumbar internal fixation surgery (21). And Chen et al. found that 17.5% of patients had poor wound healing in their study of patients with type II diabetes (22). However, in this study, only 1.5% of patients (8/547) experienced poor wound healing, and the proportion of such poor wound healing was completely underestimated. In this study, patients who underwent lumbar spine surgery were generally discharged on the 7th day after surgery, while postoperative wound stitches are usually removed 12–14 days after surgery. It is understood that some patients have poor wound healing after suture removal, but did not come to our hospital for treatment. This missing data is the reason for the low incidence of poor wound healing in patients in this study. For patients with poor wound healing, obese patients accounted for 87.5% (7/8). The obese patients induce a chronic low-grade inflammatory state through increased release of adipokines, cytokines, and chemokines from excess adipose tissue. The chronic low-grade inflammation is thought to contribute to a dampened immune response during the inflammatory phase of wound healing leading to delayed wound healing (23). At the molecular biology level, research has also demonstrated this viewpoint. In obese individuals, skin-resistant cells lead to wound non healing by reducing the generation of cytokines and growth factors and increasing the generation of IL-17 (24). Although leptin secreted by adipocytes promotes fibroblast proliferation, differentiation, and vascular regeneration through the fibroblast growth factor-2 pathway (25). However, there are currently few studies reporting the exact effects of leptin on wound healing diseases in clinical practice. Moreover, obesity in patients can negatively impact wound healing through several mechanisms, including alterations in capillary function, reduced levels of growth factors, and the formation of poor-quality granulation tissue (23). The impact of obesity on symptom recurrence has been confirmed in many literatures. Jiang et al. and Luo et al. found that obesity is a risk factor for recurrence after percutaneous endoscopic lumbar disc herniation surgery (26, 27). Compared with normal weight controls, obese patients experience an increase in intervertebral disc pressure during most daily activities, leading to accelerated intervertebral disc degeneration and studies have demonstrated that obesity is associated with an increased risk of adjacent segment degeneration following lumbar fusion surgery for degenerative lumbar disease (28).

Another independent factor leading to postoperative symptom recurrence in patients is the duration of preoperative symptoms. Previous studies have explored whether the duration of preoperative symptoms is related to postoperative outcomes. Wu, Hu et al. found that patients with preoperative symptoms lasting longer than one year had adverse outcomes, whether it was in the early or middle and late postoperative period and predicted that the rate of reoperation increased with the prolongation of preoperative symptom duration (29). In this study, we found that preoperative symptom duration lasting no less than six months was an independent risk factor for postoperative dissatisfaction. The recurrence rate of postoperative symptoms in patients increases with the duration of preoperative symptoms. We have two speculations, first of all, the longer duration of preoperative symptoms is related to the longer duration of nerve root compression, which may lead to irreversible damage to the nerve root (30). Secondly, Wang et al. have reported (28) that the duration of preoperative pain is related to adjacent segment degeneration after lumbar spine surgery, and one of the reasons for symptom recurrence is a series of back and leg pain caused by adjacent segment degeneration. However, studies have shown that postoperative rehabilitation training can effectively relieve pain and improve quality of life, and early rehabilitation training could enhance results in terms of pain and disability, as well as reduce the risk of symptom recurrence (31).

Another possible complication after lumbar spine surgery is the formation of epidural hematoma. According to reports, 0.02% −4.6% of patients developed epidural hematoma after surgery (32, 33), which is consistent with the results of this study, where 0.73% (4/547) of patients developed epidural hematoma. When analyzing the risk factors for epidural hematoma, univariate analysis found that hypertension and the amount of wound drainage were related to the occurrence of epidural hematoma. However, after conducting binary logistic analysis, no independent risk factors for epidural hematoma were found, which may be related to the low incidence of epidural hematoma and the limitation of study sample size. Wang et al. found in their retrospective study on 9,258 patients underwent posterior lumbar decompression surgery for lumbar spinal stenosis that multilevel procedures, postoperative systolic blood pressure, previous spinal surgery and abnormal coagulation are independent risk factors for epidural hematoma (34). Another protective factor for epidural hematoma is tranexamic acid, the beneficial effect of tranexamic acid on reducing blood loss in lumbar spine surgeries has already been confirmed by numerous meta-analyses (35, 36). Moreover, studies have shown that the use of tranexamic acid significantly improves the occurrence of postoperative hematoma in patients (37). The above factors can provide reference for clinical physicians.

The last and most serious postoperative complication is wound infection. In this study, the incidence of wound infection was 0.7% (4/547), and it was influenced by the small sample size and low infection rate. No risk factors were found in both single and multiple factors. However, previous studies have shown that high BMI, diabetes, long term use of corticosteroid, long operation time, and cerebral fluid leakage were independent risk factors for surgical site infection (38). Another study has also obtained similar findings, such as obesity, hypoalbuminemia, and drinking history were identified as independent risk factors (39). In this study, all four patients with wound infections were caused by improper postoperative care, which resulted in urine contamination of the wound while urinating in bed, further leading to wound infection. After culturing with wound secretions, E. coli was cultured in all wounds. We attribute the wound infections of these 4 patients to certain reasons, although they are not fully validated in this study, which may be the reason why their risk factors were not identified in this study. Fortunately, after the second debridement surgery, the patient's wounds all reached Grade A healing.

There are some limitations to this study. Firstly, as a single-center retrospective study, the baseline characteristics of patients and institutional treatment practices may introduce selection bias, potentially limiting the generalizability of our findings to broader populations. Secondly, this study lacks Bonferroni correction for multiple comparisons in univariate analysis, which may increase the risk of Type I errors. However, this potential issue was effectively addressed through multicollinearity testing and meticulous selection of variables for multivariate analysis. Additionally, a conservative significance threshold (*P* < 0.05) was employed for the inclusion of variables in the final model. Thirdly, the relatively low prevalence of rare complications constrained the statistical power of the multivariate analyses, which may have resulted in false-negative findings regarding associations with epidural hematoma or infection. Future studies with larger sample sizes or multicenter collaborations are necessary to address this limitation. Fourthly, future multicenter prospective studies with larger sample sizes and diverse patient populations are essential to validate our results and explore the general applicability of risk factors identified in this study. Fifthly, surgeries were performed by a team of 3 experienced spine surgeons at our institution, all of whom adhered to standardized TLIF protocols. Surgeon-specific were not analyzed in this study due to the retrospective design, which may introduce confounding effects on postoperative outcomes. Finally, the diagnosis of osteoporosis may be subject to some inaccuracies, primarily due to the low frequency of bone density testing among patients with lumbar disc herniation. Despite this limitation, meaningful conclusions can still be drawn. In the future, further prospective dual energy X-ray examination should be conducted to further verify the reliability of the conclusions.

## Conclusion

This study indicates that transforaminal lumbar interbody fusion surgery can safely and effectively treat lumbar disc herniation. Some factors are closely related to postoperative complications. Obesity and preoperative pain duration lasting no less than 6 months are associated with postoperative symptom recurrence. And obese patients are more likely to lead to poor wound healing. Adequate preoperative communication is particularly important for patients with these risk factors.

## Data Availability

The original contributions presented in the study are included in the article/supplementary material, further inquiries can be directed to the corresponding authors.
